# A Nexus for Gene Expression—Molecular Mechanisms of Spt5 and NusG in the Three Domains of Life

**DOI:** 10.1016/j.jmb.2012.01.031

**Published:** 2012-03-16

**Authors:** Finn Werner

**Affiliations:** RNAP Laboratory, Institute of Structural and Molecular Biology, Division of Biosciences, University College London, Gower Street, London WC1E 6BT, UK

**Keywords:** RNAP, RNA polymerase, DSB, double-stranded DNA break, LUCA, last universal common ancestor, TEC, transcription elongation complex, ops, operon polarity suppressor, transcription, RNA polymerase, Spt4/5, NusG, evolution

## Abstract

Evolutionary related multisubunit RNA polymerases (RNAPs) transcribe the genomes of all living organisms. Whereas the core subunits of RNAPs are universally conserved in all three domains of life—indicative of a common evolutionary descent—this only applies to one RNAP-associated transcription factor—Spt5, also known as NusG in bacteria. All other factors that aid RNAP during the transcription cycle are specific for the individual domain or only conserved between archaea and eukaryotes. Spt5 and its bacterial homologue NusG regulate gene expression in several ways by (i) modulating transcription processivity and promoter proximal pausing, (ii) coupling transcription and RNA processing or translation, and (iii) recruiting termination factors and thereby silencing laterally transferred DNA and protecting the genome against double-stranded DNA breaks. This review discusses recent discoveries that identify Spt5-like factors as evolutionary conserved nexus for the regulation and coordination of the machineries responsible for information processing in the cell.

## Graphical Abstract

**Figure:**
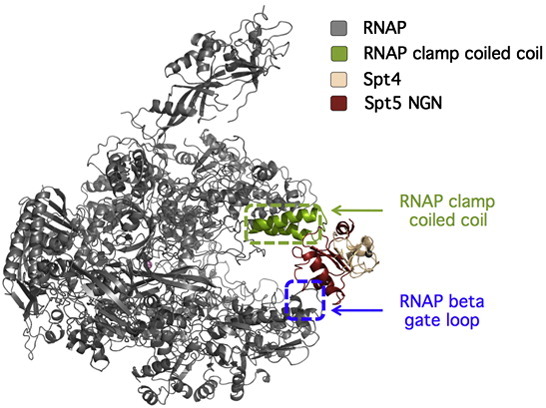

**Highlights:**

► Spt5–NusG is the only universally conserved transcription factor in the three domains of life. ► Highly versatile multifunction factor. ► Core function is to enhance transcription processivity. ► KOW domains serve as recruitment platform for broad range of factors. ► Instrumental in promoter proximal stalling in eukaryotes. ► Recruits chromatin remodelling and RNA processing factors in eukaryotes. ► Couples transcription and translation in prokaryotes. ► Recruits rho termination factor in bacteria.

## Introduction—A Conserved RNAP Regulated by Divergent Factors

Life is strictly dependent on the faithful replication of the genetic information and its expression through the synthesis of RNA and protein. This is reflected in the evolutionary conservation of the machineries that carry out these functions and in their regulation. Replication, transcription, and translation are not independent of another but are coordinated in a dynamic fashion that adjusts the needs of one with the yield of the other, and overall enables maximum energy efficiency and protection of genome integrity.

**Fig. 1:**
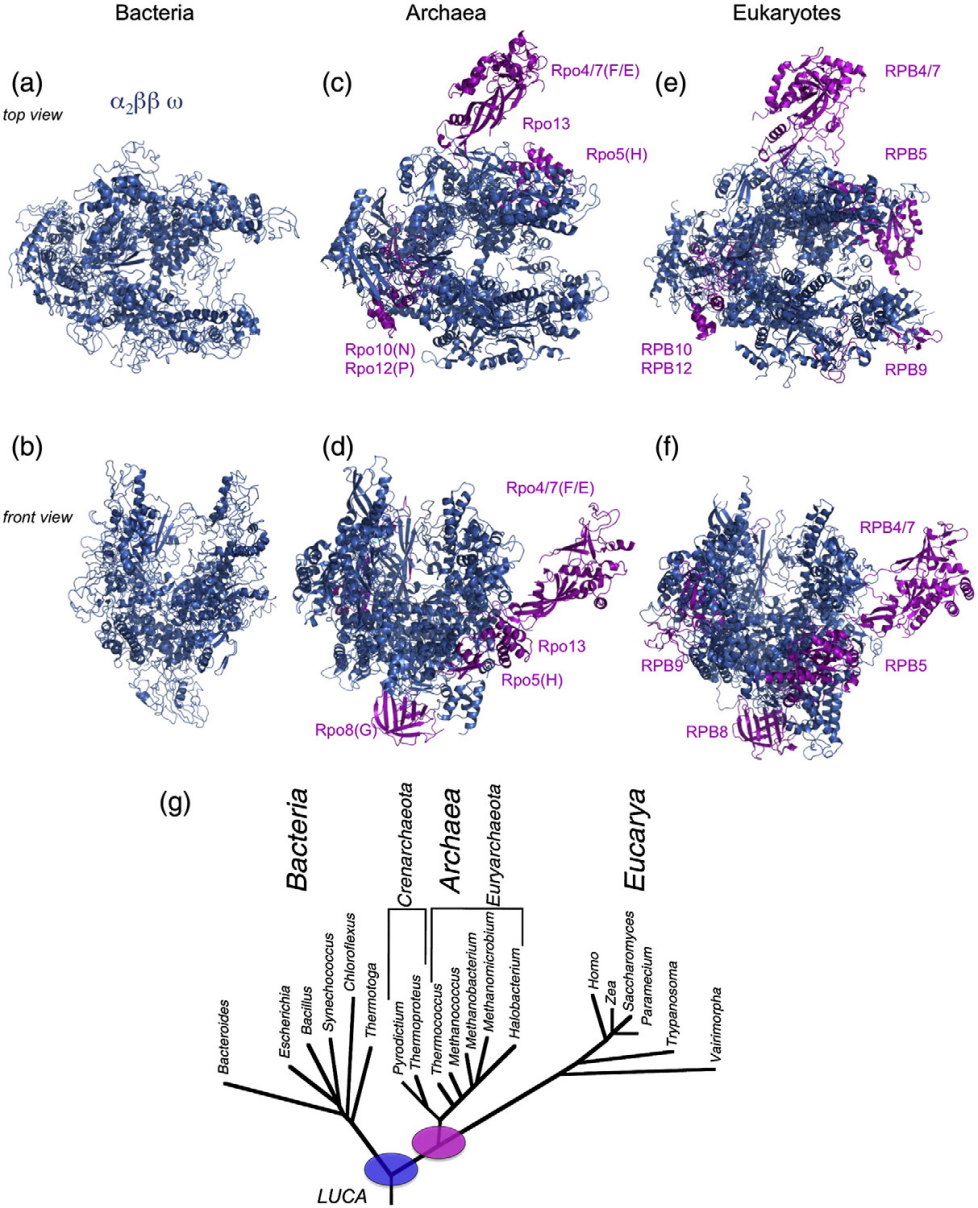
Structure of multisubunit RNAPs in the three domains of life. Representative RNAP structures are shown in top views (a, c, and e) and in front views (b, d, and f): bacterial [a and b; Protein Data Bank (PDB) ID 1I6V], archaeal (c and d; PDB ID 2WAQ), and eukaryotic (e and f; PDB ID 1NT9) RNAPs. The universally conserved core subunits are shown in blue, and the subunits specific for archaeal and eukaryotic RNAPs are highlighted in magenta. (g) The universal Tree of Life; the blue circle indicates that ancestral versions of the core RNAP subunits were present in the LUCA of all life, and the magenta circle indicates that the archaeo–eukaryotic subunits were present before the split of the archaeal and eukaryotic domains of life.

Transcription of all cellular genomes is carried out by multisubunit RNA polymerases (RNAPs), all of which are derived from one ancestral enzyme present in the last universal common ancestor (LUCA) (Fig. 1).^1,2^ This evolutionary relationship is reflected in the homology of RNAP subunit sequence and structure, overall RNAP ternary architecture, and molecular mechanisms by which RNAPs transcribe RNA in a DNA-template-dependent manner^3,4^ (Fig. 1 and Table 1). Interestingly, the same conservation does not extend to the basal transcription factors that assist RNAP during the transcription cycle.^2,5^ For example, whereas all RNAPs face the similar mechanistic challenges during transcription initiation (such as RNAP recruitment, DNA strand separation, initiation of RNA polymerisation, and promoter escape), the bacterial and archaeal/eukaryotic RNAPs utilise nonhomologous factors to facilitate these processes (Table 1). The bacterial RNAP utilises sigma factors, whereas eukaryotic RNAPII uses the basal factors TBP (*T*ATA *b*inding *p*rotein) or TFIID [TBP plus TAFs (*T*BP-*a*ssociated *f*actors)], TFIIA (*t*ranscription *f*actor *IIA*), TFIIB, TFIIE, TFIIF, and TFIIH.^6,7^ The archaeal RNAP is a streamlined version of the RNAPII system and utilises TBP, TFB (homologous to TFIIB), and TFE (homologous to TFIIE alpha).^8^

The fact that bacteria and archaea/eukaryotes make use of nonhomologous factors for initiation strongly suggests that the ancestral RNAP of LUCA did not utilise any of these factors but rather initiated transcription either factor-independently, or aided by a factor that subsequently was lost in evolution.^2^ There is a precedence for the former mechanism since (evolutionary unrelated) single subunit RNAPs (e.g., bacteriophage T7 RNAP) can initiate transcription by directly recognising the promoter DNA in a sequence-specific manner.^9^

During elongation, multisubunit RNAPs frequently pause and move in a retrograde direction along the template by backtracking, which can regulate transcription and contribute to its fidelity, but also be detrimental to productive transcription.^10–12^ Backtracked complexes are rescued by transcript cleavage factors that associate with RNAP and retune its active site, which results in a cleavage of the transcript and a new RNA 3′ terminus competent for catalysis and elongation.^11,13–15^ Similar to the initiation phase of transcription, the challenges that RNAPs need to overcome during elongation are identical, but the transcript cleavage factors that solve the problem are not homologous.^16^ In bacteria, Gre factors stimulate transcript cleavage, and in archaea/eukaryotes, TFIIS/TFS fulfil this function. Gre and TFIIS/TFS are not homologous and adopt different structures; however, they interact with RNAP in the same manner by inserting two juxtaposed acidic residues into the active centre through the NTP entry pore.^17,18^ The lack of *bona fide* homology suggests—despite the compelling similarity of the mechanism—that the LUCA RNAP did not utilise any of the factors.

During termination of transcription, the stable elongation complex has to dissociate in order to release the transcript and template and make RNAP available for the next round of transcription. The elongation complex has to undergo substantial conformational changes during termination (such as an opening of the RNAP clamp) that are not necessarily energetically favourable. In bacteria, this energy is provided by RNA secondary structure formation by Watson–Crick base pairing of intrinsic terminators or by the rho helicase by virtue of ATP hydrolysis.^19,20^ Termination by eukaryotic RNAPII is more complex and involves both polyadenylation of the transcript and an exonuclease (Xrn2/Rat1) that, despite being unrelated to rho, translocates along the RNA towards the RNAP and terminates transcription reminiscent of rho.^21,22^ Eukaryotic RNAPIII and archaeal RNAP are able to terminate transcription independently of RNA secondary structures or factors.^23–26^ Thus, similar to the transcription initiation and elongation cleavage factors, none of the termination factors are evolutionary conserved across the three domains of life.

In summary, it appears that a highly conserved RNAP system is almost exclusively aided by evolutionary nonrelated transcription factors. One important exception to this observation is Spt5/NusG, and the key functions that this factor have in gene expression might explain its universal evolutionary conservation.^27^

## Structure and Organisation of Spt5-Like Factors and Their Complexes with RNAP

**Fig. 2:**
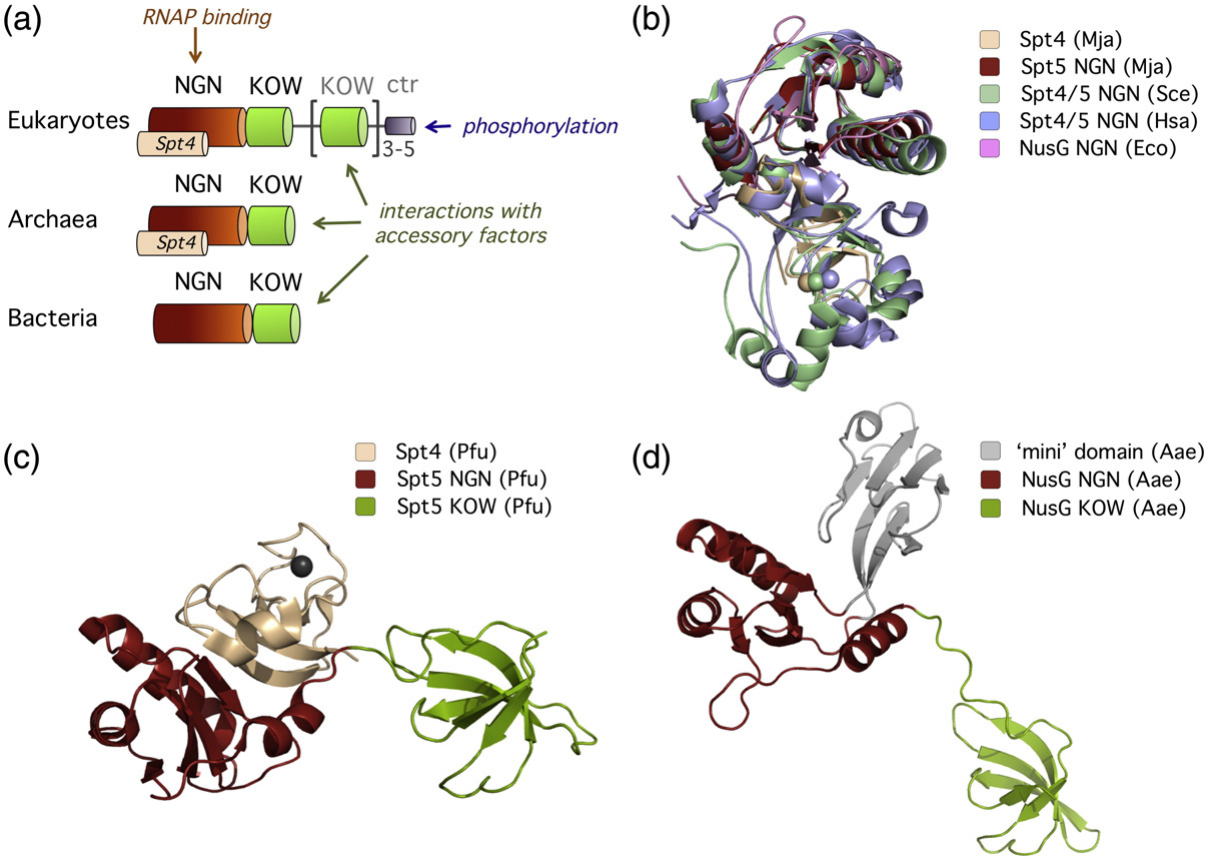
Structure and domain organisation of Spt5-like factors. Organisation of Spt4/5 and NusG (a). Spt5 consists of an NGN domain (highlighted in firebrick red) and one or more KOW (*K*yrpidis, *O*uzounis, *W*oese) domains (green), only eukaryotic Spt5 contains two C-terminal repeats (ctr). Eukaryotic and archaeal Spt5 form a complex with Spt4 (wheat); the zinc ion coordinated by Spt4 is illustrated as a sphere. (b) A structural alignment of Spt4/5 NGN from archaea (*Methanocaldococcus jannaschii*, Mja) and eukaryotes (*Homo sapiens*, Hsa; *S. cerevisiae*, Sce) and the NusG NGN domain from bacteria (*E. coli*, Eco) prepared using VMD (http://www.ks.uiuc.edu/Research/vmd/). The structure of Mja Spt4/5NGN was solved using a dimeric complex of Spt4 (wheat) and Spt5 NGN (firebrick red), and the Sce (mint green) and Hsa (light blue) Spt4/5 NGN structures were solved by crystallising a fusion protein of Spt4 and Spt5. (c) The X-ray structure of Spt4/5 from *P. furiosus* (Pfu; PDB ID 3P8B) and (d) the X-ray structure of NusG from *Aquifex aeolicus* (Aae; PDB ID 1NPR) that contains a “mini”-domain (coloured light grey) inserted into the NGN domain at a position similar to Spt4.^30^ The mini-domain is not present in all bacterial NusG variants.

**Fig. 3:**
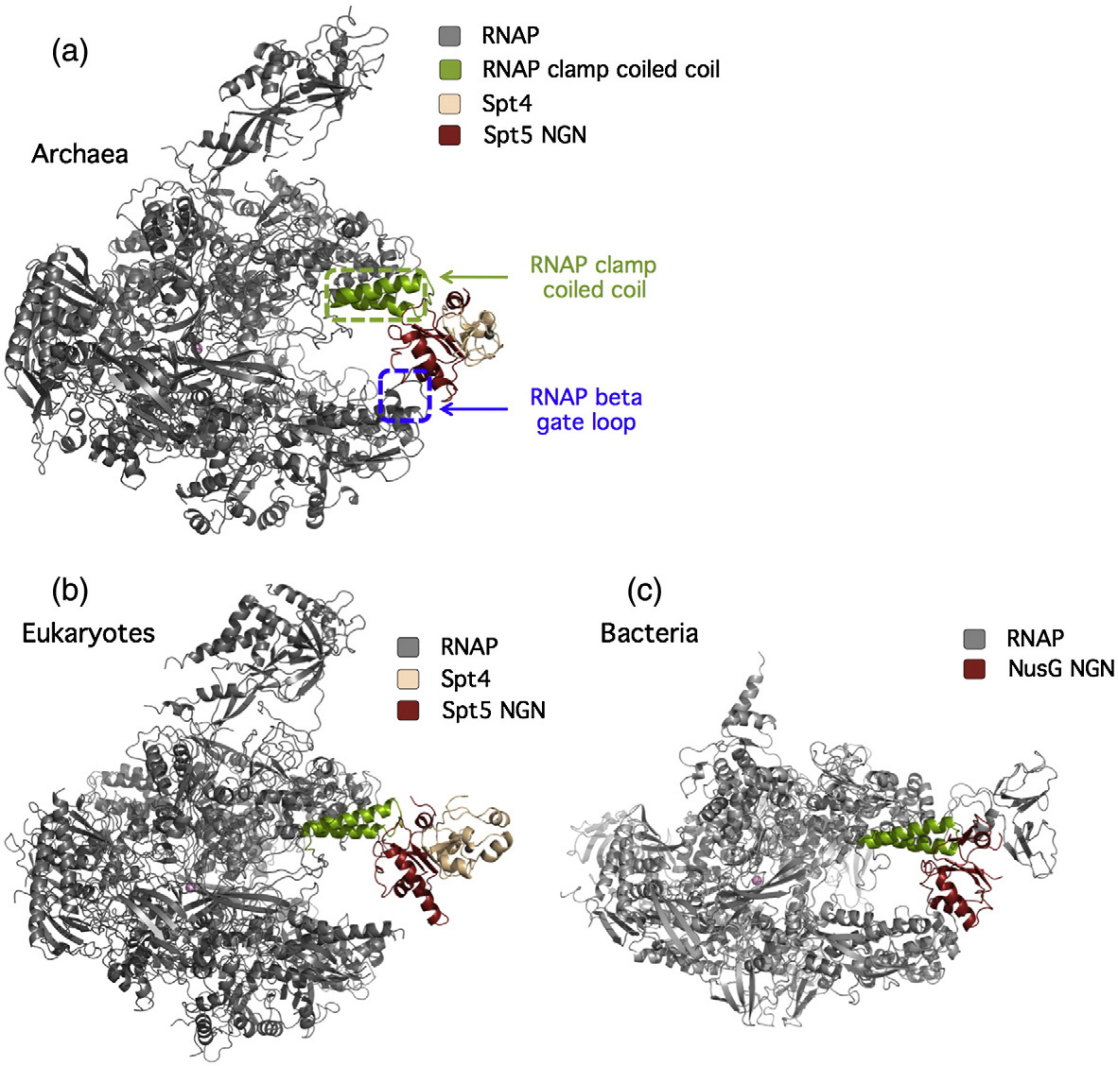
Structure of RNAP–Spt4/5 complexes in the three domains of life. (a and b) Models of RNAP–Spt4/5 complexes in archaea and eukaryotes, respectively; (c) a model of the RNAP–NusG complex in bacteria. The Spt5 NGN domain is located across the DNA binding channel by interacting with the RNAP clamp coiled coil (also known as clamp helices) on one side of the cleft and with the beta gate loop on the opposite side (highlighted with blue broken rectangles). The X-ray structure of a complex encompassing the archaeal RNAP clamp and Spt4/5 NGN from *P. furiosus* was fitted into the structures of RNAPs (modified from Ref. 34). The Spt5 NGN domain is highlighted in firebrick red; Spt4, in wheat. In the model of the eukaryotic complex, the Spt5 NGN domain does not appear to make contacts with the wall of the DNA binding channel opposite to the clamp because the beta gate loop in the parental RNAPII structure is disordered.

**Fig. 4:**
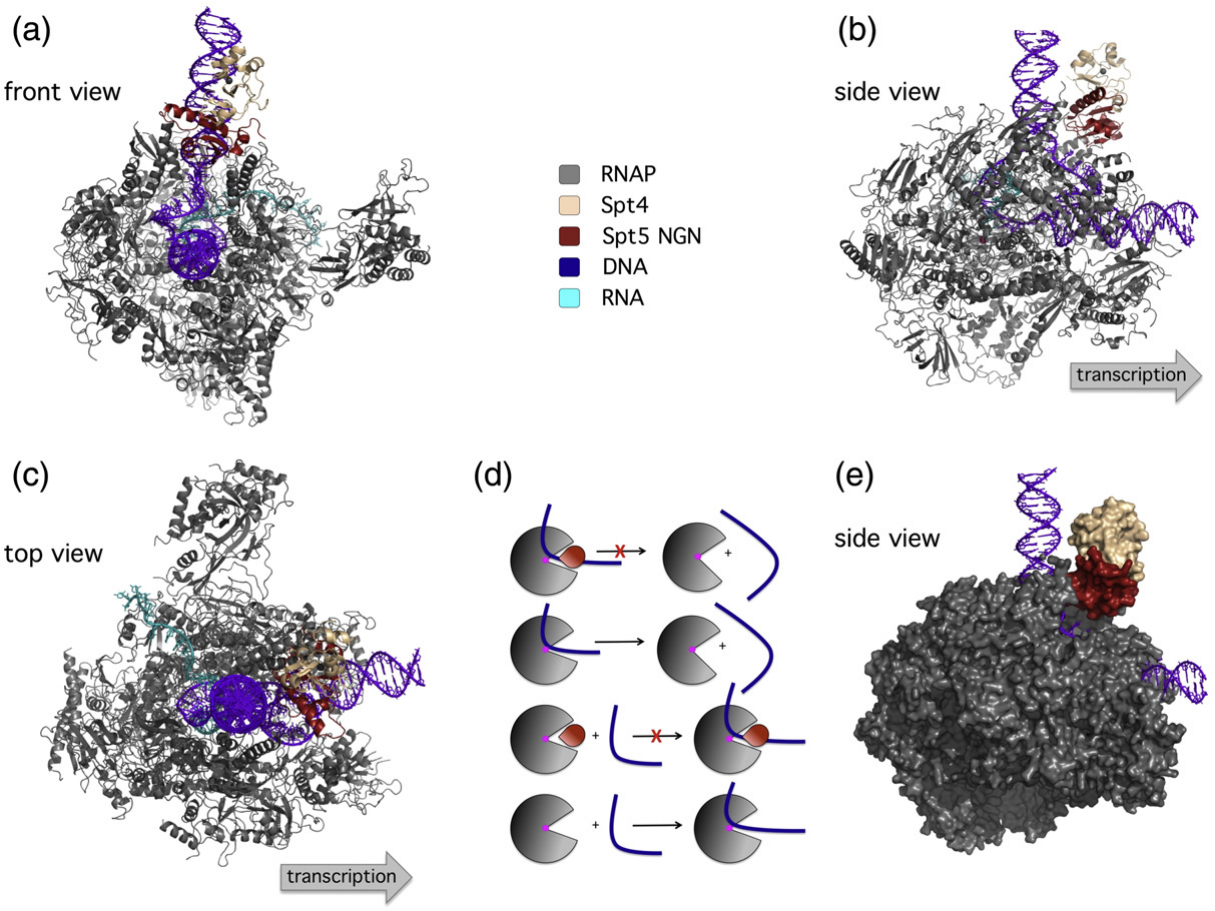
Spt4/5 locks the template in the DNA binding channel of RNAP in the TEC. Front (a), side (b and e), and top (c) views of the eukaryotic RNAPII–Spt4/5 TEC. This model was built by combining structural information of the archaeal RNAP clamp–Spt4/5 complex with the X-ray structure and single-molecule fluorescence resonance energy transfer data from the yeast RNAPII–DNA–RNA elongation complex^36,37^ (modified from Ref. 34). (d) The Spt4/5 complex (red wedge) locks the DNA–RNA hybrid and the DNA strands forming the transcription bubble into the active site, it thereby denies both dissociation and association of the DNA template (blue line) from the RNAP. As a result, when Spt4/5 associates with the RNAP–DNA–RNA elongation complex, it increases its stability; by contrast, when Spt4/5 associates with free RNAP, it suppresses nonspecific DNA binding. (e) A surface representation of the complex that emphasises how the template is locked into the RNAP [same orientation as in (b)].

Eukaryotic and archaeal Spt5 and their bacterial homologue NusG display an extensive sequence and structural homology and associate with their cognate RNAPs via evolutionary conserved binding sites.^27^ The organisation of Spt5-like proteins is modular, and all factors encompass an NGN (*N*us*G N*-terminal) domain and one (bacteria and archaea) or multiple (eukaryotes) KOW domain(s)^27–29^ (Fig. 2a). The NGN domain interacts with RNAP and stimulates its processivity,^27,28,31^ whereas the KOW domains serve as a recruitment platform for accessory factors (Table 2).^28,32^ NusG from bacteria is a monomeric transcription factor. Spt5 from eukaryotes and archaea forms a heterodimeric complex with Spt4, a small protein that stabilises the Spt5 NGN domain structure but, otherwise, has an unknown function.^27^ The evolutionary conservation of Spt5 and NusG is apparent from their amino acid sequence but becomes even more prominent when comparing the structures of the NGN domains (Fig. 2).^27,28,33^ Mutational analysis of the Spt5 and NusG NGN domains have identified a conserved hydrophobic depression that functionally interacts with the RNAP clamp coiled coil (“CC”, also referred to as clamp helices “CH” in bacteria) on one side of the DNA binding channel.^27,28,31^ The X-ray structure of a complex between a recombinant fragment of the *Pyrococcus furiosus* RNAP clamp with Spt4/5 confirms this binding site.^34^ As shown for the bacterial NusG paralogue RfaH, NGN domains also make contact with the RNAP beta gate loop (“betaGL”), which is located opposite to the clamp CC across the DNA binding channel^35^ (Fig. 3). Via these two interactions, the NGN domains of Spt5-like factors (i) lock the DNA–RNA hybrid into the active site and (ii) separate the upstream and downstream portions of the template (Fig. 4). A cryo-electron microscopy structure of the entire archaeal RNAP–Spt4/5 complex confirms that the NGN domain of Spt5 and, by inference, its homologues NusG and RfaH span across the DNA binding channel of RNAP.^38^

## Molecular Basis of the Processivity Function of Spt5-Like Factors

Spt5-like factors bind to the RNAP clamp, a structurally well conserved flexible module that closes over the DNA binding channel by a rotation of up to 30° relative to the RNAP core. The position and possibly movement of the clamp can be altered by the archaeal/eukaryote-specific RNAP stalk (consisting of the Rpo4/7 and RPB4/7 subunits; Fig. 1c–f), which has been hypothesised to play a role during the loading of the template into the active site (open complex formation) and which is important to maintain high transcription processivity.^39^ The translocation/elongation mechanism of RNAPs is facilitated by the bridge helix and trigger loop in the active site.^40^ Since both elements are connected to the inside of the clamp, even a slight reposition of the latter can have substantial impact on the elongation properties of the transcription elongation complex (TEC);^41^ that is, Spt5-like factors could alter the catalytic properties of RNAP via an allosteric mechanism.^42^

By binding across the DNA binding channel of RNAP, Spt4/5 (and NusG and RfaH) has the potential to affect RNAP in two opposite ways (Fig. 4). When Spt4/5 associates with elongating RNAPs, it could deny the *dissociation* of RNAP-bound DNA and could thereby increase the stability of the elongation complex and stimulate transcription processivity.^38,43^ When Spt4/5 associates with free RNAP, it could deny *association* of promoter DNA with the DNA binding channel of RNAP, prevent RNAP recruitment, and thereby repress transcription (Fig. 4). The stimulatory property for transcription processivity of RNAP by NusG in bacteria and Spt4/5 in archaea is well documented,^27,28^ and a recent article also supports the second, inhibitory, mechanism. Preincubation of Spt4/5 with archaeal RNAP efficiently inhibits RNAP recruitment and transcription initiation in a minimal *in vitro* transcription system.^44^

### The first swap: initiation factor TFE and elongation factor Spt4/5

The archaeal RNAP requires only two basal transcription factors for promoter-directed transcription, TBP and TFB.^8^ TBP recognises the TATA element of the promoter and bends the DNA by approximately 90°;^45^ this DNA–TBP complex is recognised by TFB, which in turn recruits RNAP and transcription initiation commences. In contrast, the Spt4/5–RNAP complex cannot be recruited to the DNA–TFB–TFB complex, and transcription initiation is repressed.^44^

### How is this repression overcome in order to assure efficient transcription in the presence of Spt4/5 in the cell?

The answer to this question is the transcription initiation factor TFE. TFE is homologous to TFIIE alpha in the eukaryotic RNAPII system^46^ and of C82 in RNAPI.^47^ TFE associates with RNAP and stabilises the open transcription initiation complex^48,49^ by two mechanisms involving an allosteric effect on the RNAP clamp and by interacting with the nontemplate strand of the promoter.^44,50^ In addition, TFE can be cross-linked downstream of promoter sequences, which suggests that it can be retained on RNAP elongation complexes after promoter escape.^50^ However, the binding site of TFE or the molecular mechanisms underlying its activities have remained obscure until recently. Grohmann *et al.* used a Förster resonance (fluorescence resonance energy transfer) system to identify the TFE binding site on RNAP in the complete archaeal transcription initiation complex.^44^ Both the TFE winged helix domain and the Spt5 NGN domain interact with the RNAP clamp coiled coil.^27,44^ The two binding sites are overlapping, and correspondingly, Spt4/5 and TFE compete for RNAP binding. Importantly, the relative binding affinities of the factors are context dependent: TFE prevails over Spt4/5 in the initiation complex, whereas Spt4/5 prevails over TFE in the elongation complex.^44^ As a result, TFE can efficiently prevent the inhibitory effect of Spt4/5 on transcription initiation.^44^

### What is the function of the transcription factor swapping during transcription?

**Fig. 5:**
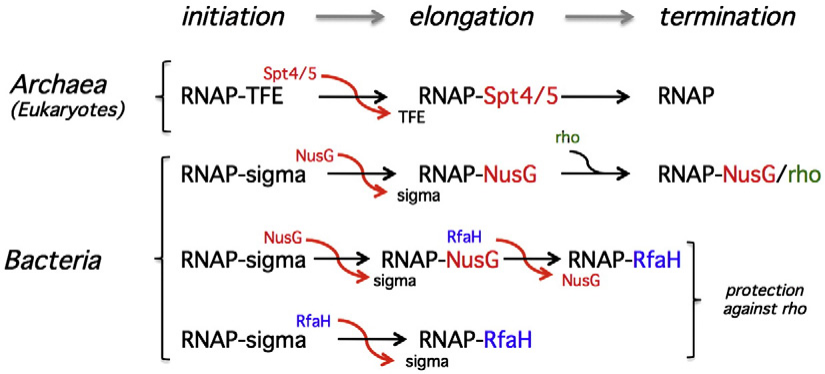
Transcription factor swapping during the transcription cycle. In the archaeal and eukaryotic systems, the binding sites for the initiation factor TFE (TFIIE alpha) and the elongation factor Spt4/5 overlap on the RNAP clamp in mutual exclusive manner. During promoter escape or early elongation, Spt4/5 displaces TFE from the RNAP. In perfect analogy, the bacterial sigma initiation factor can be displaced *in vitro* by the Spt5 homologue and paralogue NusG and RfaH, respectively. NusG is able to recruit the rho factor, which results in transcription termination. In contrast, RfaH could also displace NusG *in vivo*, which protects the elongation complex against the recruitment of rho and termination and thereby facilitates the expression of distal genes.

All RNAPs bind DNA in a nonsequence-specific manner, which can critically quench or reduce the concentration of “free” RNAP available for promoter-directed transcription. Association of RNAP with Spt4/5 has the potential to reduce this effect, and since the repression by Spt4/5 is negated by TFE at the promoter, the overall outcome is an increase in promoter specificity of RNAP. In addition, or alternatively, the transition between the initiation and elongation phases of transcription—referred to as promoter escape—may be aided by the factor swap. One of the intrinsic conflicts during transcription initiation is the apparent need for high-affinity interactions between RNAP and promoter DNA to ensure efficient RNAP recruitment and the ability to readily release the promoter-bound RNAP necessary to enable a high firing rate of the promoter.^2^ This is made possible by the binding mode of initiation factors to RNAP where multiple contacts are made with a combined high affinity.^51^ Conformational changes in the initiation complex during promoter escape disrupt the individual interactions in a stepwise manner. The distinct conformational states of RNAP in the initiation and elongation complexes may be stabilised by associated transcription factors, and the energy barriers for the changes between the conformational states can be lowered by association with transcription factors. Thus, Spt4/5 displacing TFE could stimulate promoter escape (Fig. 5). Alternatively, Spt4/5 could ensure that RNAP-bound TFE during early elongation is efficiently displaced and replaced by Spt4/5, resulting in a high-processivity RNAP–Spt4/5 TEC. Whole genome occupancy profiling of RNAPII and transcription initiation and elongation factors in *Saccharomyces cerevisiae* has revealed a relatively sharp transition between initiation and elongation factors (including Spt4/5) at approximately 150 bp downstream of the transcription start site.^52^ Whereas this position is too far downstream to qualify as promoter escape, it supports the notion of an efficient swap between initiation and elongation factors. Interestingly, the bacterial transcription initiation factor sigma, despite not being evolutionary related to TFE, also interacts with the RNAP CH.^53,54^ Sigma can, like TFE, remain associated with RNAP during early elongation and induce pausing.^55^ Importantly, NusG and RfaH, like Spt5, interact with the RNAP clamp CH, and both can compete with sigma for RNAP binding^56^ (Fig. 5).

In summary, the mechanism of competition binding between initiation and elongation factors for RNAP is universally conserved in evolution.

### The second swap: elongation and termination (co-) factor NusG and RfaH

In the bacterial system, NusG is an essential general transcription factor that has pleiotropic effects on transcription elongation.^28,57–59^ NusG is a component of the antitermination complex that converts RNAP into a termination-resistant form, which is able to read through early intrinsic termination signals in, for example, ribosomal RNA (rrn) operons and during late gene expression of the bacteriophage lambda.^60^ NusG stimulates processivity by suppressing transcription pausing (also referred to as antipausing), but it also acts in concert with rho to facilitate termination.^10,59,61,62^ rho itself interacts with RNAP and is recruited to the TEC via physical interactions with the NusG KOW domain (Figs. 5 and 7). Thus, NusG association with RNAP enhances elongation but also directly contributes to termination. The whole genome occupancy profile of NusG demonstrates that it is bound to TECs on nearly all genes, protein encoding and noncoding transcription units.^63^ NusG appears to be recruited to genes in a stochastic fashion downstream of the promoter, and it is overrepresented on longer operons. Whether the latter is due to the fact that TEC on long genes have more opportunities to recruit NusG or whether NusG association with RNAP enables the TEC to transcribe longer genes is unclear.^63^ RfaH is a nonessential sequence-specific paralogue of NusG that induces the expression of genes downstream of the operon polarity suppressor (ops) DNA element.^64^ Transcription of the ops sequence triggers recruitment of RfaH to TEC and directs the transcription of a small subset of operons involved in bacterial virulence.^65^ On the sequence level, RfaH and NusG are closely related, and this homology extends to the structural level with respect to their NGN domains.^66^ However, whereas the NusG C-terminus is a typical KOW domain entirely consisting of beta sheets, the RfaH C-terminal domain in full-length RfaH is a radically different all-alpha helical fold.^66^ RfaH, despite being present in the cell at much lower concentration than NusG, excludes NusG from binding RNAP once the TEC has transcribed through the ops element.^67^ Whole genome occupancy profiles demonstrate that RNAP and NusG peaks overlap just downstream of the promoter on the *rfb* operon—indicative of NusG recruitment immediately after transcription initiation where the swap of sigma and NusG has occurred.^35^ A second swap is executed with a sharp transition at approximately 1 kb when NusG is replaced by RfaH. This reduces transcription termination facilitated by rho by at least two independent mechanisms, a stimulation of transcription processivity by converting RNAP into a high-processivity (pause-resistant state) TEC similar to NusG and by preventing the recruitment of rho via the NusG KOW domain.^35^ The latter is demonstrated by the occupancy profile of rho on the *rfb* operon. The net outcome of the second swap (between NusG and RfaH) in the very early stages of transcription elongation is the efficient transcription of genes distal to the ops sequence.

In summary, both NusG and RfaH interact with the RNAP using a conserved mechanism, but the two factors have opposing regulatory roles. NusG in concert with rho represses the expression of “foreign” genes (see below), while RfaH inhibits rho action on RNAP and thereby enhances the expression of “foreign” genes such as the *rfb* operon.^35,67^

## A Paradigm Shift—Eukaryotic Spt4/5 and Promoter Proximal Pausing

In eukaryotes, Spt4/5 is associated with RNAPII on most, if not all, genes;^52^ Spt5 is an essential gene in *S. cerevisiae*, whereas Spt4 is dispensable at permissive temperatures.^68^ This is in good agreement with results from the archaeal system, which demonstrate that Spt4 has a stabilising effect on the Spt5 NGN domain but is not strictly required for its binding to RNAP nor its stimulatory effect on processivity.^27^ Eukaryotic Spt5 variants contain four to six copies of KOW domains (in comparison to one in archaea and bacteria) and two C-terminal repeat regions that are subject to phosphorylation but not required for cell viability in yeast.^69,70^ The additional KOW domains serve as binding or recruitment sites for a plethora of factors including NELF (*n*egative *el*ongation *f*actor) and kinases such as P-TEFb (*p*ositive *t*ranscription *e*longation *f*actor *b*) and Bur-1/Bur-2, factors that are involved in chromatin remodelling such as Spt6 and FACT, and RNA processing factors such as the mRNA capping enzyme and the cap methyl transferase^32^ (Table 2).

**Fig. 6:**
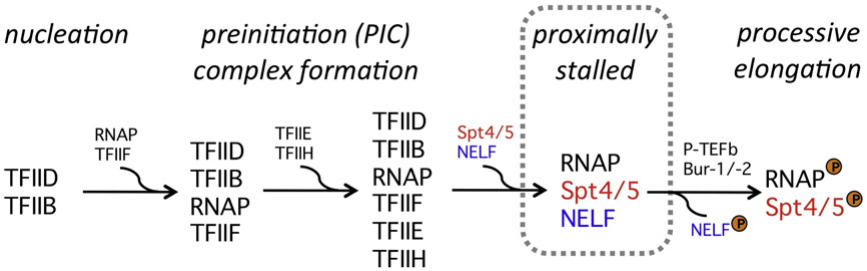
Spt4/5 facilitates promoter proximal pausing in metazoans. Binding of TFIID to the TATA element of a eukaryotic class II promoter triggers a recruitment cascade that results in the initiation of transcription. However, the association of Spt4/5 and NELF with RNAPII in the early elongation complex leads to promoter proximally stalled elongation complexes. Activation of kinases including P-TEFb and Bur-1/Bur-2 leads to the phosphorylation of the RNAPII C-terminal domain, Spt4/5, and NELF, which releases the stalled complexes and induces robust gene expression.^74^ Many class II genes in metazoans harbour promoter proximally stalled initiation complexes, and it is possible that this mechanism operates on a global level.^75^ Phosphorylation events are highlighted with an orange “P”.

The classical paradigm of transcription control—regulation is chiefly controlled by the recruitment of the RNAPII to the promoter—was established using the yeast transcription machinery. Early observations of the regulation of the hsp70 heat shock gene in a metazoan system, *Drosophila melanogaster*, suggested that its transcription was *not* regulated at the level of RNAPII recruitment. Rather, TECs were paused (also referred to as stalled or poised) proximal to the promoter approximately 40 bp downstream of the transcription start site.^71,72^ The complexes were catalytically competent but not able to penetrate into the downstream gene. Signalling events induced by heat shock lead to the activation of the kinase P-TEFb that phosphorylates multiple components of the elongation complex including Spt4/5, NELF, and RNAPII^73^ (Fig. 6). This results in the release of the poised elongation complexes and robust expression of the heat shock genes. The regulatory advantage of these mechanisms is a fast induction achieved by uncoupling of RNAP recruitment and transcription.^76^ In the last 5 years, it has become apparent that many, if not most, genes in metazoans harbour promoter proximally stalled RNAPII and are therefore likely to be subject to this regulation.^74,75^ Promoter proximal stalling could have other functions than regulation such as mRNA quality control and coupling between transcription and mRNA processing.^77^ A delay of elongation complexes could thus allow the recruitment of RNA modification enzymes such as the capping enzyme, and elongation would commence only if the capping is completed. Both Spt4/5 and NELF are required for promoter proximal pausing, but the molecular mechanisms are still not understood. Spt4/5 is a key candidate for directly inhibiting RNAP activity because its binding to RNAP alters the DNA-binding properties of the enzyme and possibly induces conformational alterations in the active centre (see above) that, in theory, could pause transcription elongation.^44^ The significance of the position of the paused complexes at + 40 is unclear, even though it seems compelling that human RNAPII via the RPB4/7 complex is interacting with approximately 40 nucleotides of the transcript^78^ and that the recruitment of eukaryotic Spt4/5 to RNAPII is enhanced, if not dependent on, interactions between the Spt5 KOW domains and the RNA transcript.^79^

In summary, the Spt4/5 complex is associated with many, if not all, class II genes in eukaryotes, and it plays a pivotal role for a eukaryote-specific mechanism that regulates the processivity of transcription—promoter proximal stalling. In addition, like its prokaryotic counterpart, it is involved in the recruitment of accessory factors that regulate gene expression, alter the availability of the chromatin template, and are involved in the processing of the nascent transcript. The function of Spt4/5 in eukaryotes is not restricted to class II genes since Spt4/5 also directly associates with RNAPI and modulates its processivity both positively and negatively.^80,81^

## Prokaryotes—NusG-Like Factors Connect RNAP, Ribosomes, and rho

**Fig. 7:**
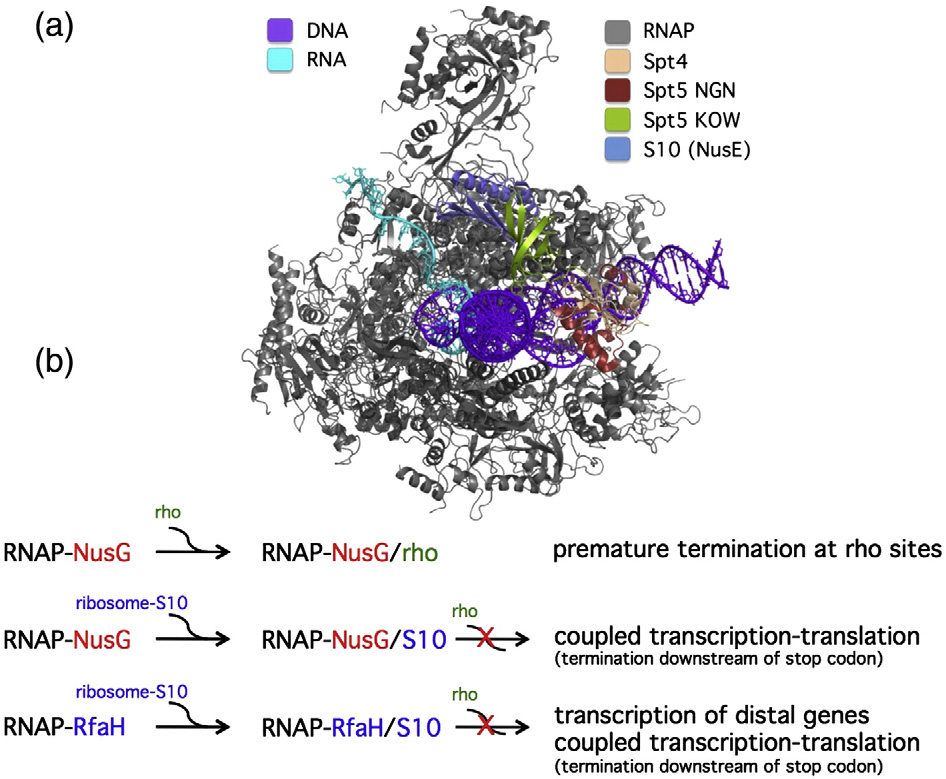
Transcription and translation are coupled via NusG in prokaryotes. (a) Structural model of the RNAP–Spt4/5–S10 elongation complex. The S10 protein (light blue) of the 30S ribosomal subunit forms a complex with the KOW domain (green) of Spt5, and thus, the ribosome is ideally positioned to interact with the mRNA template (cyan). This model was built by combining the X-ray structure of the NusG KOW–S10 complex from *E. coli* (PDB ID 2KVQ), the structure of the archaeal Spt4/5 complex from *P. furiosus* (PDB ID 3P8B), and a model of the RNAPII–Spt4/5 NGN complex from *S. cerevisiae*.^34^ The components are colour coded according to the key in the figure. The RNA interacts with the RNAP stalk (Rpo4/7 or RPB4/7) during transcription elongation, but the RNA species included in the X-ray structure (PDB ID 1NT9) was too short to observe this interaction. (b) Mechanisms of coupled transcription–translation. The NusG KOW domain interacts with the rho factor and ribosomal protein S10 in a mutual exclusive manner. RNAP–NusG elongation complexes are able to recruit rho, which can lead to (pre-) mature termination at rho termination sites. Ribosomal protein S10 can bind to the NusG KOW domain and thereby prevent the recruitment of rho. Thus, the efficient coupling of transcription and translation (i.e., of RNAP and ribosome) prevents premature termination. Following translation termination, the ribosome dissociates from the transcript and makes the NusG KOW domain accessible for rho binding, which promptly leads to transcription termination. Likewise, the NusG paralogue RfaH can recruit ribosomes, protect against rho, and facilitate the expression of distal genes.

In both prokaryotic domains, bacteria and archaea, transcription and translation are coupled.^82^ In bacteria, the rates of transcription and translation are correlated over a broad range of growth rates, which keeps the ratio between the two elongation rates at 3 (three nucleotides for every one amino acid).^83^ As a result, the transcription yield is correlating with the translation needs on different genes and during varying growth rates. In effect, the elongation rate of transcription is influenced by the presence of ribosomes and their elongation rate of translation; the average elongation rate of RNAPs can be increased by cotranslating ribosomes, possibly because the latter reduce pausing and backtracking of RNAP. Ribosomes whose elongation rates have been decreased by antibiotic action in turn cause a decrease in transcription elongation rates of RNAP by virtue of their intrinsic propensity to pause and backtrack. In both situations, little mRNA is exposed and available for recognition by rho; that is, the gene is protected against rho-dependent termination.^83^ These phenomena could be explained by the motions of two molecular motors, one of which uses the product of the other as template (mRNA). Recently, it has emerged that the NusG KOW domain physically interacts with ribosomal protein S10 (first characterised as antitermination factor NusE) and that this interaction could tether elongating ribosomes and RNAPs^84^ (Fig. 7a). In principle, this interaction could also enhance ribosome recruitment during translation initiation. Since S10 competes with rho for the binding to the NusG KOW domain, the coupling of transcription and translation protects the TEC against rho-dependent termination^84^ (Fig. 7). Translation is terminated at the 3′ end of operons, and S10 dissociates from RNAP-bound NusG, which makes the KOW domain available for the recruitment of rho and subsequent efficient transcription termination.^84^ In summary, Spt5-like factors have the potential to coordinate transcription and translation in prokaryotes, bacteria and archaea.

## The Silencing of Foreign DNA and Maintenance of Genome Stability

Bacterial genomes contain a considerable load of horizontally transferred or “foreign” DNA including prophages, insertion sequence elements, and K-island clusters.^85^ Many of these elements can be deleted to obtain reduced genome strains, which sometimes have beneficial biotechnological properties and different requirements for essential genes than their parental strains.^86^ In the synthetic *Escherichia coli* strain MDS42, 14% of the genome of the parental strain MG1655 has been deleted. In contrast to MG1655, NusG (and NusA) is not essential for cell viability in MDS42.^87^ Moreover, deletion of the lambdoid prophage rac from MG1655 is sufficient to make NusG dispensable.^87^ Considering that MDS42 has a dramatically increased (∼ 10^4^×) resistance to the highly selective inhibitor of the rho termination factor (bicyclomycin), it is possible that the function of NusG that makes it indispensable in the wild-type strain is due to its role as a rho cofactor for rho.^83^ In other words, NusG in conjunction with rho silences rac gene expression, which otherwise would kill the cell. A whole genome expression analysis reveals that bicyclomycin treatment predominantly upregulates the expression of foreign DNA, which suggest that rho represses its expression.^87^ Since rho to a large extent relies on NusG as cofactor, NusG may play an important role for the silencing of foreign DNA on a global level.

Transcription and replication occur simultaneously, and collisions between the TEC and the replication fork not only are inevitable but also occur on a regular basis.^88,89^ Since the replisome moves at a greater speed (20×) than the TEC, even codirectional collisions happen frequently, and these can lead to double-stranded DNA breaks (DSBs). Two recent articles show that rho-dependent termination protects against DSBs by removing arrested elongation complexes.^90,91^ In particular, the DSBs caused by codirectional collisions were dependent on backtracked TECs.^91^ The cell has evolved a range of strategies to prevent DSBs caused by backtracked TEC. Transcription factors that improve transcription processivity and suppress backtracking and factors that reactivate backtracked TEC by transcript cleavage reduce the occurrence of DSB.^91^ Efficient coupling of transcription and translation, that is, of RNAP and ribosomes, suppresses backtracking and reduces DSB.^91^ Transcription termination factors such as rho and its cofactors that remove backtracked and arrested TEC from the genome also protect against DSB. The common denominator of all these strategies is NusG: it increases processivity, efficiently couples transcription and translation, and promotes termination in conjunction with rho.

## Conclusion and Perspective

Spt5-like transcription factors are universally conserved in evolution and serve multiple functions in the three domains of life.

### What is the significance of their evolutionary ancient origin, and what was the role of the Spt5 ancestor for transcription carried out by the RNAP of the LUCA?

The most basic function of Spt5-like proteins that is common to all transcription systems is the stimulation of transcription processivity carried out by the universally conserved Spt5 NGN domain. The exact composition of early genomes is unclear; they could have consisted of either DNA or RNA, either in single-stranded or in double-stranded form. The catalytic centre of multisubunit RNAPs can utilise single-stranded DNA as template^23^ and, under special circumstances, RNA as template for transcription. For example, RNAPII can transcribe the hepatitis virus delta RNA genome, and bacterial RNAP uses the noncoding 6S RNA regulator as template for RNA-dependent RNA polymerisation.^92,93^ However, the processivity of transcription on these templates is severely impaired, possibly because the interactions between RNAP and single-stranded DNA or RNA are weaker as compared to double-stranded DNA. Likewise, ancestral versions of multisubunit RNAPs were likely capable of using nonduplex DNA as template.^94^ The ancestral version of Spt5 might have been crucial for robust RNAP function using these early “poor” genome templates, and thus, Spt5 could have provided a crucial selective advantage for its host. Experimental evidence supports this notion, since archaeal Spt4/5 strongly enhances the processivity of its cognate RNAP using single-stranded template DNA strand.^27^ Not unlike the RNAP stalk,^23^ the Spt5 ancestor may have played a crucial role for the expression of long genes and, during evolution, even have “permitted” an increase in gene or operon length and thereby assisted an increase in the complexity of the genetic repertoire of the organism. Once established as an integral component of TECs, other functions including the recruitment of transcription termination factors (such as rho in bacteria), the coupling of RNAPs to ribosomes (in prokaryotes), and nascent RNA processing (in eukaryotes) might have emerged over time due to the advantageous location of Spt5 in the TEC: across the DNA binding channel of RNAP, proximal to the nascent transcript, and bound to RNAP in a reversible manner. All recruitment sites for exogenous factors are located in the Spt5 KOW domain(s). The expansion of the number of KOW domains in evolution (e.g., one in prokaryotes, four in yeast, and six in humans) seems in-line with the notion that they facilitate more elaborate, later additions to the functionality of Spt5-like factors.

## Figures and Tables

**Table 1:**
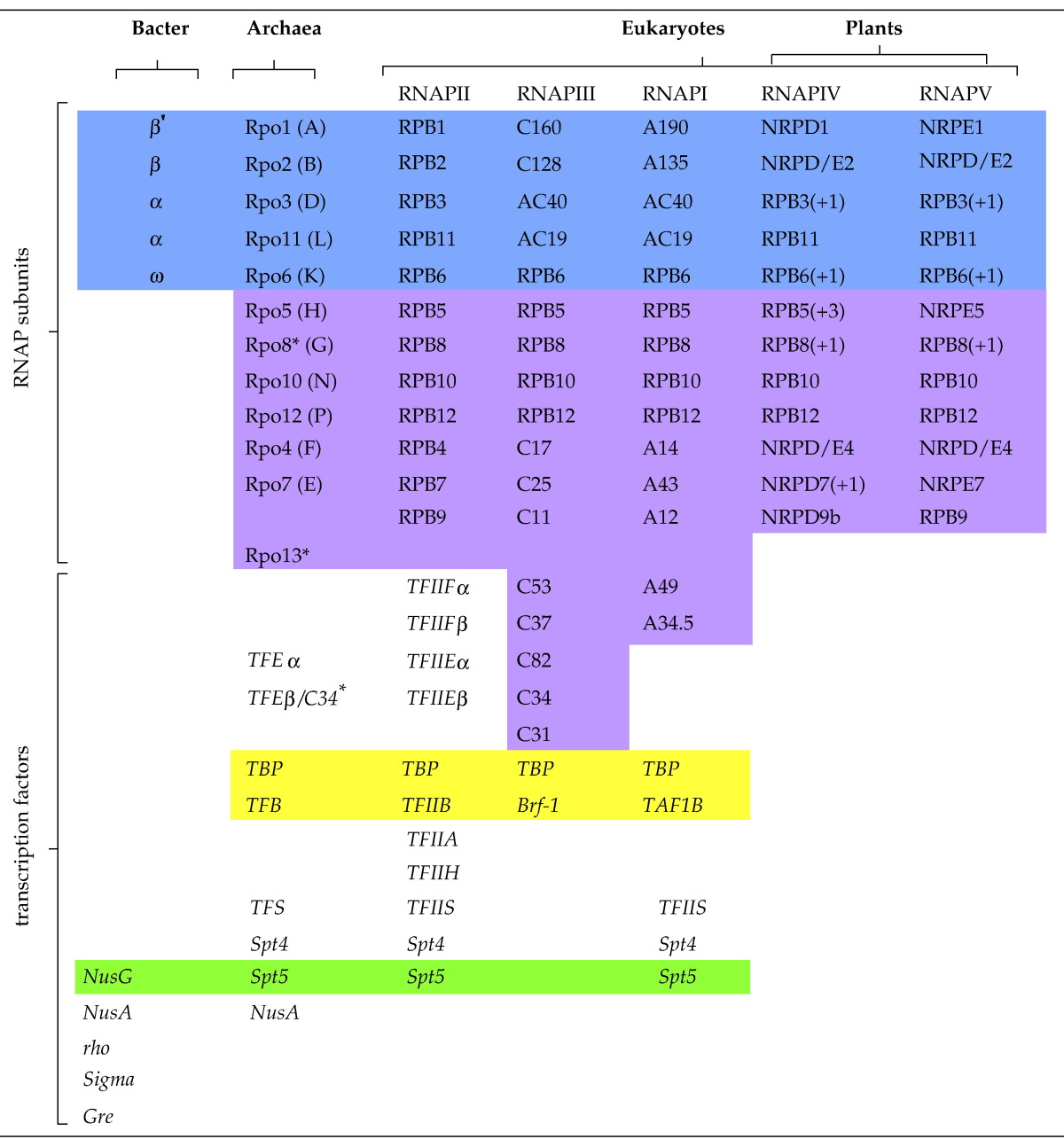
Evolutionary conservation of RNAP subunits and transcription factors in the three domains of life

Homologous polypeptides are summarised in rows; RNAP subunits and transcription factor discussed in the text are organised in columns. The universally conserved RNAP subunits are highlighted in blue, and the subunits specific for archaea and eukaryotes are highlighted in magenta. Note that some of the subunits of RNAPI and RNAPIII are paralogous to RNAPII transcription factors. The only RNAP-associated transcription factor that is universally conserved in all three domains of life is Spt5/NusG (highlighted in green). The combination of TBP and TFB-like proteins is required for transcription of the archaeal and all eukaryotic RNAPs (highlighted in yellow). Polypeptides marked with asterisks (⁎) are only present in some species, and the number in brackets after the RNAPIV and RNAPV subunits indicates multiple genes.

**Table 2:** The KOW domains of Spt5 and NusG interact with a plethora of factors

Domain	Factor	Sequence	Interactor	Function
Bacteria	NusG	Nonspecific	S10/ribosome rho	Coupling of transcription and translation termination; silencing of foreign DNA
	RfaH	ops	S10/ribosome	
Archaea	Spt5	Nonspecific	S10/ribosome	Coupling of transcription and translation
Eukaryotes	Spt5	Nonspecific	NELF P-TEFb, Bur-1/Bur-2 FACT, Spt6 capping enzyme Cap methyl transferase	Promoter proximal pausing Phosphorylation of RNAP, NELF, and Spt4/5 Chromatin remodelling RNA processing

In prokaryotes, the KOW domain of NusG (and Spt5) interacts with ribosomal protein S10 and thereby physically connects RNAPs and ribosomes. It also interacts with the termination factor rho and functionally acts as its cofactor. In eukaryotes, the multiple KOW domains of Spt5 facilitate interactions with RNAPI and RNAPII, with the transcript and with a wide range of accessory factors involved in the regulation of transcription and transcript maturation.
